# Overcoming Implementation Barriers of Concurrent Treatment for Eating Disorders and Posttraumatic Stress Disorder: Two Novel and Feasible Approaches

**DOI:** 10.3390/bs15060749

**Published:** 2025-05-30

**Authors:** Kathryn Trottier, Sara Bartel, Aaron Keshen

**Affiliations:** 1 Eating Disorders Program, Toronto General Hospital, University Health Network, Toronto, ON M5G 2C4, Canada; 2 Department of Psychiatry, University of Toronto, Toronto, ON M5T 1R8, Canada; 3 Eating Disorders Program, Nova Scotia Health, Halifax, NS B3S 0H6, Canada; sara.bartel@nshealth.ca (S.B.); aaron.keshen@nshealth.ca (A.K.); 4 Department of Psychiatry, Dalhousie University, Halifax, NS B3H 2E2, Canada

**Keywords:** eating disorders, posttraumatic stress disorder, integrated treatment, concurrent treatment, feasibility

## Abstract

Eating disorders (EDs) and posttraumatic stress disorder (PTSD) frequently co-occur and share a functional relationship. Evidence suggests benefits of integrated and/or concurrent treatment; however, implementation is hindered by clinician training burden and the challenges of delivering two treatments simultaneously. This paper explores two novel and feasible approaches to addressing ED-PTSD. The first is a clinician-guided cognitive behavioural workbook intervention delivered concurrently with ED treatment. It involves psychoeducation, addresses dissociation, and encourages approach (versus avoidance) practices. The second involves combining Written Exposure Therapy (WET) with ED treatment at both outpatient and day hospital levels of care. Both interventions have a low training burden and are feasible in routine clinical practice, making concurrent approaches available to those who need them.

## 1. Introduction

Eating disorders (EDs) and posttraumatic stress disorder (PTSD) commonly co-occur. The estimated pooled prevalence rate is 24.59% for PTSD within ED samples and 20.05% for EDs in PTSD samples (when weighted by study quality; Ferrell et al., 2022). A bidirectional and functional relationship between the disorders has been hypothesized, wherein ED and PTSD symptoms and psychopathology are connected and reinforce one another (Trottier et al., 2016; Mitchell et al., 2021). For example, traumatic experiences such as sexual assault can contribute to a negative body image, and hunger and satiety can be disrupted because of the emotional numbing, avoidance, and hyperarousal symptoms of PTSD. ED behaviours often appear to serve as avoidant coping methods for PTSD symptoms, wherein food restriction, binge eating, and purging facilitate avoidance of trauma-related memories and feelings, as well as decrease arousal (e.g., Brewerton, 2007). When individuals with EDs work toward establishing a pattern of regular and adequate eating in treatment and stop ED behaviours such as purging, they may experience increased distress due to trauma-related memories and emotions and ultimately revert to ED behaviours as a means of coping. Findings from mediational and network analysis research are consistent with the notion of a bidirectional and functional relationship between the disorders (Mitchell et al., 2021). Importantly, systematic reviews suggests that the presence of comorbid PTSD can negatively impact ED treatment outcomes in that it predicts dropout from ED treatment and relapse following ED treatment (Convertino & Mendoza, 2023; Day et al., 2024; Liebman et al., 2025). Given the potential for PTSD to maintain ED symptoms and psychopathology, having a treatment plan that considers both diagnoses is increasingly viewed as important.

Recent guidance on the treatment of ED-PTSD suggests that sequential interventions that focus on one diagnosis at a time do not fully address maintaining mechanisms, and may therefore leave patients vulnerable to relapse (Brewerton, 2023). In contrast, concurrent (separate interventions delivered at the same time) and integrated (one intervention that addresses both disorders) treatments have a greater potential to address the reciprocal relationship between the disorders, and there is some evidence to suggest integrated approaches are preferred by patients (Trottier et al., 2022). Several concurrent and integrated treatment approaches for ED-PTSD have emerged in the literature, particularly in the context of intensive ED treatment programs. Both prolonged exposure (PE) and cognitive processing therapy (CPT) (two of the most strongly recommended PTSD interventions; APA, 2017; NICE, 2018) have been delivered concurrently with day hospital and/or residential ED treatment and were associated with significant reductions in PTSD symptoms (e.g., Brewerton et al., 2023; Claudat et al., 2022). An integrated CPT and cognitive–behavioural therapy (CBT) for ED intervention has also been developed and evaluated in uncontrolled (Trottier et al., 2017) and randomized controlled research (Trottier et al., 2022). In both studies, integrated CBT for ED-PTSD was delivered following a course of inpatient and/or day hospital ED treatment and was associated with significant improvements in PTSD symptoms. Given the timing of delivery after a full course of intensive ED treatment, further improvements in ED symptoms were not expected; that said, there was no evidence of worsening of ED symptoms associated with the integrated CBT (a worsening is often expected by clinicians; Trottier et al., 2017). Although these concurrent and integrated approaches hold promise, both CPT and PE require clinicians to have specific and time-intensive training in the protocols and are time-consuming (1–1.5 h weekly sessions for 8–12 weeks) to deliver. These factors make it difficult for concurrent or integrated treatments for ED-PTSD to be made readily available, especially given other factors limiting the accessibility of PTSD interventions in general (e.g., clinician and patient fears about treatment, financial cost, long wait times; Smith et al., 2020).

Trauma-related interventions that are highly scripted, brief, and have a low clinician training burden are arguably more feasible to deliver in the context of ED treatment. Meta-analytic data suggest that self-directed interventions for PTSD are effective, with moderate-to-large effect size reductions in PTSD symptoms (Siddaway et al., 2022; Simblett et al., 2017; see also Steubl et al., 2021). These interventions can be unguided, meaning that the patient works through the intervention on their own, or they can include clinician guidance. Brief clinician-delivered PTSD treatments are also available; one such promising intervention is written exposure therapy (WET). WET involves five 1 h sessions of guided writing exercises focused on trauma exposure. In contrast to CPT and PE, there are no between-session practice exercises. WET is a manualized treatment that is highly structured and scripted, thus requiring minimal clinician training and making it a practical option to deliver across diverse clinical settings. It is also associated with significantly less dropout than CPT and PE (Sloan et al., 2021; Sloan et al., 2022). A recent systematic review of 17 WET studies found moderate-to-large effect size improvements in PTSD symptoms, with no difference in efficacy between WET and first-line PTSD treatments such as CPT and PE (DeJesus et al., 2024).

Brief PTSD treatments, such as WET, have been implemented within treatment programs for other mental health concerns commonly comorbid with PTSD, such as substance use programs (Van Doren et al., 2025; Schumacher et al., 2023). However, research examining the use of brief PTSD interventions within ED samples has been scant and limited to case series (e.g., Emily et al., 2024; Keshen et al., 2025). In this paper, we present an overview of two novel, feasible, and sustainable approaches to addressing ED-PTSD comorbidity that have the potential to make trauma-related interventions widely available to individuals receiving treatment for their ED, namely a clinician-guided workbook intervention and concurrent or integrated delivery of WET with ED treatment.

## 2. Cognitive–Behavioural Tools to Help You Move Forward in Recovery from Your Eating Disorder and Trauma: A Clinician-Guided Cognitive–Behavioural Workbook Intervention

“Cognitive–Behavioural Tools to Help You Move Forward in Recovery from Your Eating Disorder and Trauma” is a self-directed intervention that was developed as an adjunctive intervention to Toronto General Hospital (TGH)’s ED day hospital program, which was CBT-based. Rates of co-occurring PTSD were high amongst patients in the program (over 40%; Trottier, 2020), likely related to the higher illness severity and impairment typical of individuals with co-occurring PTSD compared to those without co-occurring PTSD (Liebman et al., 2025). Clinicians had long described trauma-related symptoms as a common and persistent obstacle to patients’ ability to benefit from treatment in the day hospital program (Trottier et al., 2017). Some of the most commonly noted obstacles were dissociation that interfered with participation in individual and group sessions, as well as follow-through on out-of-program practice, avoidance of foods that were reminders of traumatic events, and increased trauma-related distress following the implementation of a regular pattern of eating and interruption of ED behaviours. Consistent with this, PTSD was found to be the only significant predictor of premature termination of the program (Trottier, 2020). Patients with co-occurring PTSD were over twice as likely to end treatment early and were particularly vulnerable to dropping out during the first four weeks of treatment, when the focus of treatment was on establishing a pattern of eating full regular meals and interrupting purging and other ED behaviours.

In cases of ED-PTSD, it was common for both clinicians and patients to feel “stuck”. “Cognitive–Behavioural Tools to Help You Move Forward in Recovery from Your Eating Disorder and Trauma” was developed to be a low-barrier, highly accessible adjunctive intervention for individuals in ED treatment who have co-occurring PTSD symptoms. The goals of the intervention were to help individuals with ED-PTSD engage with, complete, and have good outcomes from ED treatment, as well as to move them onto a path of trauma recovery through practicing approach (versus avoidance). It is important to note that the intervention was not intended as a replacement for trauma-focused interventions, and this is made clear through the psychoeducation component of the intervention.

**Figure 1:**
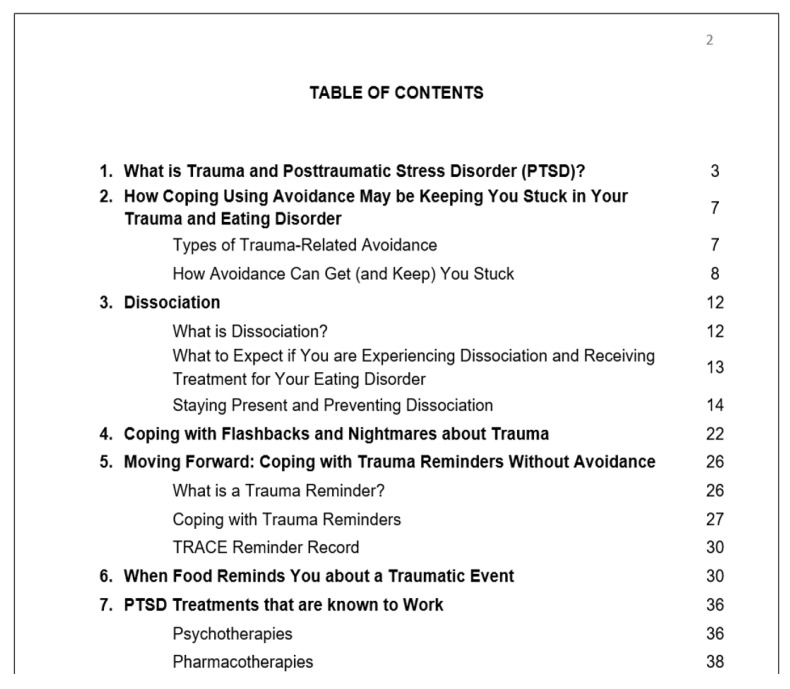
Table of Contents for “Cognitive–Behavioural Tools to Help You Move Forward in Recovery from Your Eating Disorder and Trauma”.

The intervention involves a workbook, developed to be consistent with principles of trauma-competent care (e.g., ensuring all patients receive information about trauma and its potential effects; ensuring trauma-related information and resources provided are consistent across the service) (Brewerton, 2019). In our experience, patients who presented for ED treatment were often receiving information from healthcare providers about trauma and PTSD that was not consistent with evidence-based models of PTSD recovery. It was common for patients to be treated as fragile and not capable of recovering from trauma, and to be told that evidence-based PTSD treatments were not appropriate or would not work for them. Thus, the workbook intervention provides psychoeducation about trauma, the potential for interaction between trauma-related symptoms and ED symptoms, and the process of trauma recovery consistent with evidence-based models of PTSD treatment; provides a structure, starting point, and includes practice for addressing trauma-related avoidance; addresses potentially treatment-interfering symptoms such as dissociation; and provides information about PTSD treatments that are most strongly recommended in practice guidelines (e.g., APA, 2017; NICE, 2018). Dissociation is conceptualized as an avoidant coping strategy and is addressed from a strengths-based perspective via psychoeducation, skill building (strategies to stay present), and practice. Psychoeducation is provided about conditioned responses to trauma-related reminders, and a worksheet called a TRACE record helps people to separate out current situations that are reminders of traumatic events from the past traumatic event to reduce distress and prevent escape and avoidance. The workbook underscores that it is not intended to be a treatment for PTSD and that trauma-focused interventions are the most effective treatments for trauma-related symptoms. See Figure 1 for the workbook table of contents.

### Implementation/Delivery

Within any clinical service for EDs, implementation of the intervention should start with a standardized means of identifying individuals with ED-PTSD who would likely benefit from it. Most individuals with ED-PTSD do not seek treatment for PTSD or self-identify as having PTSD. Thus, consistent with principles of trauma-competent care, a standardized approach to screening all patients for PTSD is recommended as a means of identifying patients who should be offered the intervention. A standardized measure such as the PTSD Checklist-5 (PCL-5; Blevins et al., 2015) is recommended and is a feasible method of identifying probable cases of PTSD and subthreshold PTSD. Importantly, given that ED symptoms may attenuate PTSD symptoms, many people with ED-PTSD may have symptoms that are subthreshold for a diagnosis, and these individuals are also likely to benefit from the workbook intervention. It is recommended that a clinician follows up with patients who screen positive for PTSD or subthreshold PTSD to provide feedback on the screening results and offer the workbook intervention. When offering the workbook, it is important to provide a strong clinical rationale, and, if the individual is reluctant to accept, to seek to understand their hesitation and normalize any urges to avoid while encouraging them to approach. Currently, at TGH, all patients (across inpatient and outpatient levels of care) are screened for PTSD symptoms at the start of treatment using the PCL-5 and all positive screens are followed up on by the patient’s individual CBT therapist, who offers the workbook. All of TGH’s services are cognitive–behavioural; thus, the therapeutic approach in the workbook is consistent with the approach utilized in ED treatment. In our experience, the great majority of patients agree to receive the intervention. In the rare case that someone declines, the therapist may revisit it with the patient if trauma-related symptoms appear to be interfering with ED treatment.

It is recommended that the workbook be delivered with some form of clinician guidance. At TGH, the patient’s individual CBT therapist guides the patient through the psychoeducation workbook by assigning the patient to read and complete specific chapters and then meeting with the patient to discuss the readings, worksheets, and practice. Patients typically receive three to five individual guidance sessions of 20–30 min that focus on providing encouragement, clarifying the material, helping to consolidate learning and insights, problem-solving barriers to completing the readings, worksheets, and practice, and providing positive reinforcement for any efforts to approach (versus avoid). Typically, the intervention is offered in addition to treatment-as-usual, shortly after the individual starts ED treatment. However, on occasion, if trauma-related symptoms are known to be an obstacle to engaging in ED treatment (i.e., based on clinical history), it is delivered beforehand. The number of guidance sessions delivered is in the higher end of the range for individuals who are experiencing trauma-related symptoms that are, or are known to, actively interfere with ED treatment (e.g., dissociation, high distress, and/or escape and avoidance in response to trauma reminders).

To date, the workbook intervention has been delivered with clinician guidance to well over 300 patients across TGH’s outpatient, day hospital/intensive outpatient, and inpatient levels of care. Unfortunately, the potential benefits of the intervention have not yet been formally evaluated using research methods. However, as part of a quality improvement process, patients who received the intervention as part of the initial implementation were asked to provide feedback via a self-report survey. Patients with ED-PTSD who received the workbook intervention reported the following:

- A total of 64% indicated that the PTSD screening process helped them to disclose their trauma;
- A total of 91% reported having a better understanding of trauma and PTSD;
- A total of 73% reported receiving new information;
- A total of 73% reported a better understanding of how trauma can be related to mental health problems other than PTSD, such as EDs, substance misuse, suicidality, and self-injury;
- A total of 100% of patients reported that they perceived that their trauma-related avoidance was contributing to their ED;
- A total of 82% reported that they had learned new coping strategies;
- A total of 91% reported that they were now trying to reduce their trauma-related avoidance;
- A total of 73% reported being hopeful they could recover from their trauma;
- A total of 82% reported they were ready to participate in an evidence-based trauma-focused treatment;
- A total of 91% reported that they would participate in an evidence-based trauma-focused treatment if they could access it.

From clinician and program operations perspectives, the sense of “stuckness” that was pervasive in both patients and clinicians before implementing the workbook intervention is no longer evident in clinical rounds and case consultation discussions, and trauma-related dissociation and avoidance rarely occur to the extent that they are ED treatment-interfering. Clinicians are now well equipped to address these issues in ED treatment and, overall, patients are highly receptive to the rationale and approach in the workbook. Although the intervention has had a clearly observable impact on improving the ED treatment experience of individuals with ED-PTSD, it will be important to evaluate whether the intervention improves PTSD symptoms (and if so, the magnitude of the effect) through program evaluation and/or research methods to better understand its potential benefits and limitations.

## 3. Concurrent and Integrated Written Exposure Therapy and Eating Disorder Treatment

### 3.1. Concurrent WET and ED Inpatient and Day Hospital Program

Our team at Nova Scotia Health has recently published a case series evaluating the feasibility and preliminary effectiveness of administering WET concurrently with intensive ED treatment (Keshen et al., 2025). Nine patients with comorbid ED-PTSD diagnosed through clinical interviews participated in the study. The cohort, predominantly white females, with four identifying as non-binary, had an average age of 30.67 years. Their index traumas were primarily sexual trauma, with one case of combat trauma. Participants received WET once weekly for 5 weeks during inpatient or day hospital treatment. The decision to incorporate WET into either the inpatient or day hospital program was guided by the point at which PTSD was first identified during the course of treatment. The inpatient program focused on medical stabilization and weight restoration to a target body mass index (BMI) of 18–20 kg/m^2^. After inpatient treatment, most patients transitioned to a group-based, 10-week day hospital program emphasizing CBT principles, meal support, and relapse prevention.

Outcome measures were collected pre- and post-WET and included the PCL-5 (Blevins et al., 2015) to assess severity of PTSD symptoms, the Eating Disorder-15 (ED-15; Tatham et al., 2015) to evaluate eating-related cognitions and behaviours, and BMI. All participants experienced decreases in PCL-5 scores, with the mean score dropping from 57.33 (SD = 11.35) before WET to 44.78 (SD = 13.50) after WET. Six patients achieved clinically significant improvements, defined as a reduction of 10 or more points, and one participant’s score dropped below the diagnostic threshold for PTSD. However, despite these clinically meaningful reductions in symptom severity, the average post-treatment score remained above the diagnostic threshold for PTSD, perhaps highlighting the limitations of this brief protocol. Regarding ED symptoms, most patients demonstrated stability or slight improvement in ED-15 scores, with no instances of symptom deterioration. Similarly, BMI values either increased or remained stable, aligning with the goals of the ED treatments. These findings are important, as ED clinicians often express concerns that addressing PTSD in ED treatment could exacerbate ED symptoms or hinder treatment progress (Trottier et al., 2017). It is worth noting that like the findings from integrated CBT for ED-PTSD (Trottier & Monson, 2021), concurrent WET and intensive ED treatment did not generally lead to significant improvements in ED symptoms. One possible explanation for this is a “plateau effect”, where patients—most of whom had recently completed inpatient treatment before entering the day hospital program—may have already reached a point of maximum improvement in their ED symptoms prior to beginning WET.

### 3.2. Development and Implementation of an Integrated WET and Brief CBT for ED Protocol

Building on the success of the concurrent WET and intensive ED treatment case series, we developed an integrated protocol that combines WET with Brief Cognitive–Behavioural Therapy for Non-Underweight Patients (CBT-T) to simultaneously address the mechanisms maintaining both ED and PTSD symptoms. CBT-T is a condensed, 10-session version of traditional 20-session CBT for EDs designed to treat EDs efficiently while maintaining the effectiveness of longer protocols (Waller et al., 2019). CBT-T focuses on reducing maladaptive eating behaviours and modifying distorted cognitions related to body image, weight, and food. The treatment emphasizes behavioural change, including early implementation of regular eating patterns, and cognitive restructuring to address the underlying beliefs driving ED pathology. By condensing the treatment to 10 sessions, CBT-T offers a time-efficient alternative that has demonstrated comparable outcomes to longer CBT formats in improving ED symptoms (Paphiti & Newman, 2023; Keegan et al., 2022).

In Session 1 of the integrated CBT-T/WET protocol, psychoeducation is provided to elucidate the bidirectional relationship between PTSD and EDs, highlighting how ED symptoms may serve as avoidance mechanisms for trauma-related distress while PTSD symptoms like hyperarousal can exacerbate disordered eating behaviours. A personalized formulation is collaboratively developed with the patient to visually map out this interplay, tailoring the explanation to their specific traumatic experiences; however, care is taken in doing this to avoid patients sharing details or the stories about their traumatic events before they can be processed using WET. WET is then integrated into Phase 3 of CBT-T, which typically addresses general emotional triggers for ED behaviours. WET is delivered according to the standard protocol over five weekly sessions, which are extended in length (1.5 h rather than 1 h) to allow adequate time for both trauma processing and ED-focused work. Following the completion of the five WET sessions, Phase 4 of CBT-T/WET may include behavioural experiments that help patients challenge trauma-related feared foods and engage in body image work, specifically addressing concerns about body shape and size that are perpetuated by PTSD symptoms.

Our team has developed a case series study protocol to evaluate the acceptability and effectiveness of the integrated CBT-T/WET intervention (Harris et al., 2024). The study includes participants who meet diagnostic criteria for bulimia nervosa, binge-eating disorder, or atypical anorexia nervosa, as well as comorbid PTSD. The primary objective is to assess the feasibility and clinical utility of the integrated treatment. Acceptability will be evaluated through qualitative interviews conducted pre- and post-intervention, and effectiveness will be measured using validated self-report instruments assessing changes in ED symptoms, PTSD symptoms, anxiety, and depression. We have met our Phase 1 recruitment goal of 10 participants, and are now moving into Phase 2, which involves conducting clinician and patient focus groups. These focus groups will draw on qualitative feedback from Phase 1 to refine and optimize the intervention for future implementation.

## 4. Discussion

This paper explores two low-intensity approaches to addressing ED-PTSD comorbidity in the context of ED treatment. Although research investigating the efficacy and effectiveness of these interventions is needed, both interventions have great potential to make integrated care accessible to all patients with ED-PTSD who are receiving ED treatment.

Importantly, the estimated clinician hours required per patient to deliver these interventions is 5 h for the WET interventions and 1.5–2.5 h for the workbook intervention. In contrast, first-line PTSD treatments like PE and CPT require 12–22.5 face-to-face clinician hours. Moreover, our brief trauma-related interventions do not require delivery by clinicians with intensive specialized training. Training for WET delivery in the Nova Scotia Health ED treatment program involves reading the manual and participating in peer case consultation led by a psychologist with CPT training. Similarly, training to deliver the workbook intervention involves reading the workbook and attending (or watching a recording of) a 1 h workshop on the material; clinicians also participate in group case consultation led by a psychologist. Both interventions have been delivered by clinicians from several different disciplines (psychiatry, psychology, nursing, social work, etc.) and involve a simple workflow. These factors (i.e., face-to-face clinician time, clinician training requirements) can have an enormous effect on feasibility of implementation in clinical practice. The minimal training and time requirements for these interventions versus first-line PTSD treatments can make the difference between being able to provide a concurrent or integrated trauma-related intervention to every patient in ED treatment with co-occurring PTSD versus not at all. This would represent a great leap forward from the current standard of care of referring patients on for sequential treatment of their PTSD, given there is evidence individuals with ED-PTSD prefer an integrated treatment approach and few patients in ED treatment actually go on to receive first-line treatment for PTSD.

This paper focused primarily on intervention factors as barriers to implementing integrated treatment approaches for ED-PTSD. However, there are important patient factors that can also be barriers to implementation. Individuals with ED-PTSD need to be ready, willing, and able to participate in trauma-related interventions. Although there are no evidence-based guidelines, it is generally recommended that individuals with significant food restriction and/or who are significantly underweight first have a period of nutritional rehabilitation in order to be able to effectively engage in the cognitive and emotional processing required for trauma-focused interventions (e.g., 4 weeks of regular eating, some weight restoration in those with a very low BMI). Additionally, individuals need to be able to manage ED and other behaviours that may serve as avoidant coping strategies and interfere with PTSD treatment (e.g., binge eating, purging, dissociation). Please see Trottier and Monson (2021) for a detailed discussion of contraindications and recommendations for when to start integrated PTSD treatment. The workbook intervention, which does not involve trauma processing and primarily aims to prevent PTSD symptoms from interfering with ED treatment, can be effectively implemented early in ED treatment and even beforehand. Given its focus on psychoeducation, skill building, and preventing avoidance, it may also help people to become ready, willing, and able to participate in trauma-focused interventions.

It is also important to consider that concurrently delivered low-intensity interventions for PTSD may not result in a complete remission of PTSD symptoms for individuals with ED. Indeed, the workbook intervention, which mainly involves psychoeducation and skills and approach practices and does not involve trauma processing, is not intended to be a replacement for first-line PTSD treatments and is not expected to result in remission of PTSD. Psychoeducation and such skills have been shown to be inadequate for addressing PTSD (e.g., Brouzos et al., 2022), and the included approach exercises are targeted at promoting effective ED treatment. With respect to the WET protocol, when it was integrated into intensive ED treatment, PCL-5 scores tended to reduce notably but remain in the range of clinical significance. In light of this, low-intensity interventions may be an appropriate first step in a stepped-care model, particularly if more intensive trauma-focused treatments are not available or are not feasible to provide to all individuals in a clinical service. In a stepped-care model, if patients do not experience PTSD remission following low-intensity interventions, first-line treatments would be recommended as a next step.

Research is needed to understand the nature and magnitude of the benefits of these interventions and to inform decisions about whether and how these brief low-intensity trauma-related interventions should be made widely available. RCTs comparing these interventions to standard care, as well as to concurrent and/or integrated first-line ED and PTSD treatments, are needed to establish their efficacy for improving ED and PTSD symptoms, preventing treatment dropout and relapse, as well as to assess their cost effectiveness. Should lower-intensity interventions be found to be beneficial, future research may help determine who would be most appropriate to start with a lower-intensity intervention as described in this paper versus a more intensive intervention (e.g., PE, CPT).

Clients holding a diverse range of identities (e.g., gender, race, sexual orientations) have received these ED-PTSD interventions in our publicly funded treatment programs. However, the interventions were not specifically tailored to address factors such as minority stress, which may influence ED or PTSD development and maintenance (e.g., Santoniccolo & Rollè, 2024; Veldhuis et al., 2023). There is opportunity for lived-experience engagement to ensure the interventions are well suited to individuals of all identities, especially considering that research suggests certain identities are associated with an increased severity of ED-PTSD symptoms. For example, research suggests individuals with EDs who are gender-diverse or who hold other LGBTQ2IA+ identities are more likely to have experienced a greater number of traumatic events, have a greater incidence of PTSD, and have more severe PTSD symptoms (Brewerton et al., 2022). Additionally, research suggests that BIPOC individuals or LGBTQ2IA+ individuals are more likely to face inequitable access to mental health care, including ED and PTSD treatments (McClendon et al., 2020; Penwell et al., 2024). By offering concurrent or integrated PTSD intervention to those accessing ED treatment, the barriers to accessing PTSD interventions are likely to be greatly reduced for these individuals.

A decade ago, the ED field was facing the dilemma of clinically recognizing that trauma-related symptoms were an obstacle to good ED treatment outcomes but not knowing how to safely, effectively, or practically help people move forward toward mental health recovery. At the time, clinicians tended to hold beliefs that patients in ED treatment were not “ready” to process their trauma, that first-line PTSD treatments would not meet the needs of these patients, and that addressing trauma in individuals with EDs was likely to lead to a host of negative side effects such as increased self-injury, substance use, suicidality, and ED symptoms (Trottier et al., 2017). There was little research available supporting the need for concurrent and/or integrated treatment of ED-PTSD and no research examining such an approach. Since then, there have been significant advances in both research and clinical practice. Research has provided support for the hypothesized functional relationship between ED and PTSD symptoms, systematic reviews have provided evidence to confirm clinician’s perspectives that trauma-related symptoms can negatively impact ED treatment engagement and outcomes, and a small but growing body of research evaluating concurrent and integrated approaches to treatment has emerged (Liebman et al., 2025). There is also evidence of significant shifts in clinical practice with several major treatment programs in the United States now routinely providing first-line PTSD treatment concurrent with intensive ED treatment (e.g., Brewerton et al., 2023; Claudat et al., 2022). However, barriers to the widespread implementation of concurrent and/or integrated first-line treatments for ED and PSTD are numerous and significant. It is our hope that other clinicians will develop additional low-barrier concurrent and/or integrated interventions for ED-PTSD, and that researchers will evaluate the efficacy and effectiveness of our interventions and others. Brief low-intensity interventions for ED-PTSD have the potential to overcome several major implementation barriers (e.g., clinician training burden, face-to-face clinician hours for delivery, workflow challenges, already long wait times) and change the standard of care for these patients, enabling them to reach their recovery goals.

## Data Availability

The original contributions presented in this study are included in the article. Further inquiries can be directed to the corresponding author(s).

## Abbreviations

The following abbreviations are used in this manuscript:

ED	Eating Disorder
PTSD	Posttraumatic Stress Disorder
PE	Prolonged Exposure
CPT	Cognitive Processing Therapy
APA	American Psychological Association
NICE	National Institute of Health and Care Excellence
CBT	Cognitive–Behavioural Therapy
WET	Written Exposure Therapy
PCL-5	PTSD Checklist-5
TGH	Toronto General Hospital
BMI	Body Mass Index
ED-15	Eating Disorder-15
CBT-T	Brief Cognitive–Behavioural Therapy for Non-Underweight Patients
BIPOC	Black, Indigenous, and People of Colour
LGBTQ2IA+	Lesbian, Gay, Bisexual, Transgender, Queer, Two-spirited, Intersex, Asexual
